# Size Distribution of Sperm Whales Acoustically Identified during Long Term Deep-Sea Monitoring in the Ionian Sea

**DOI:** 10.1371/journal.pone.0144503

**Published:** 2015-12-16

**Authors:** Francesco Caruso, Virginia Sciacca, Giorgio Bellia, Emilio De Domenico, Giuseppina Larosa, Elena Papale, Carmelo Pellegrino, Sara Pulvirenti, Giorgio Riccobene, Francesco Simeone, Fabrizio Speziale, Salvatore Viola, Gianni Pavan

**Affiliations:** 1 Dipartimento di Scienze Biologiche e Ambientali, University of Messina, Messina, Italy; 2 Istituto Nazionale di Fisica Nucleare (INFN)—Laboratori Nazionali del Sud, Catania, Italy; 3 Dipartimento di Fisica ed Astronomia, University of Catania, Catania, Italy; 4 Bioacoustics Lab, IAMC Capo Granitola, National Research Council, Torretta Granitola (TP), Italy; 5 Istituto Nazionale di Fisica Nucleare (INFN)—Bologna, Bologna, Italy; 6 Dipartimento di Fisica ed Astronomia, University of Bologna, Bologna, Italy; 7 Istituto Nazionale di Fisica Nucleare (INFN)—Roma1, Roma, Italy; 8 Centro Interdisciplinare di Bioacustica e Ricerche Ambientali (CIBRA), Dipartimento di Scienze della Terra e dell’Ambiente, University of Pavia, Pavia, Italy; Pacific Northwest National Laboratory, UNITED STATES

## Abstract

The sperm whale (*Physeter macrocephalus*) emits a typical short acoustic signal, defined as a “click”, almost continuously while diving. It is produced in different time patterns to acoustically explore the environment and communicate with conspecifics. Each emitted click has a multi-pulse structure, resulting from the production of the sound within the sperm whale’s head. A Stable Inter Pulse Interval (Stable IPI) can be identified among the pulses that compose a single click. Applying specific algorithms, the measurement of this interval provides useful information to assess the total length of the animal recorded. In January 2005, a cabled hydrophone array was deployed at a depth of 2,100 m in the Central Mediterranean Sea, 25 km offshore Catania (Ionian Sea). The acoustic antenna, named O*ν*DE (Ocean noise Detection Experiment), was in operation until November 2006. O*ν*DE provided real time acoustic data used to perform Passive Acoustic Monitoring (PAM) of cetacean sound emissions. In this work, an innovative approach was applied to automatically measure the Stable IPI of the clicks, performing a cepstrum analysis to the energy (square amplitude) of the signals. About 2,100 five-minute recordings were processed to study the size distribution of the sperm whales detected during the O*ν*DE long term deep-sea acoustic monitoring. Stable IPIs were measured in the range between 2.1 ms and 6.4 ms. The equations of Gordon (1991) and of Growcott (2011) were used to convert the IPIs into measures of size. The results revealed that the sperm whales recorded were distributed in length from about 7.5 m to 14 m. The size category most represented was from 9 m to 12 m (adult females or juvenile males) and specimens longer than 14 m (old males) seemed to be absent.

## Introduction

The Ocean noise Detection Experiment (O*ν*DE), a deep-sea multidisciplinary observatory, was installed offshore the harbour of Catania (Eastern Sicily), at 2,100 m depth. O*ν*DE was in operation from January 2005 to November 2006. The reported data analysis has been carried out in the framework of the SMO (Submarine Multidisciplinary Observatory) project [1, 2], to study the acoustic emissions of marine mammals living or transiting in the South-western Ionian Sea [3, 4]. In this work, an automatic analysis was developed and applied on a dataset acquired by the O*ν*DE underwater acoustic observatory. A subsample (2,128 five-minute long recordings) of the large dataset acquired in the year 2005 was processed to assess the size of the recorded sperm whales by measuring the structure of their acoustic signals.

### Sound Production in the Sperm Whale’s Nose

**Fig 1:**
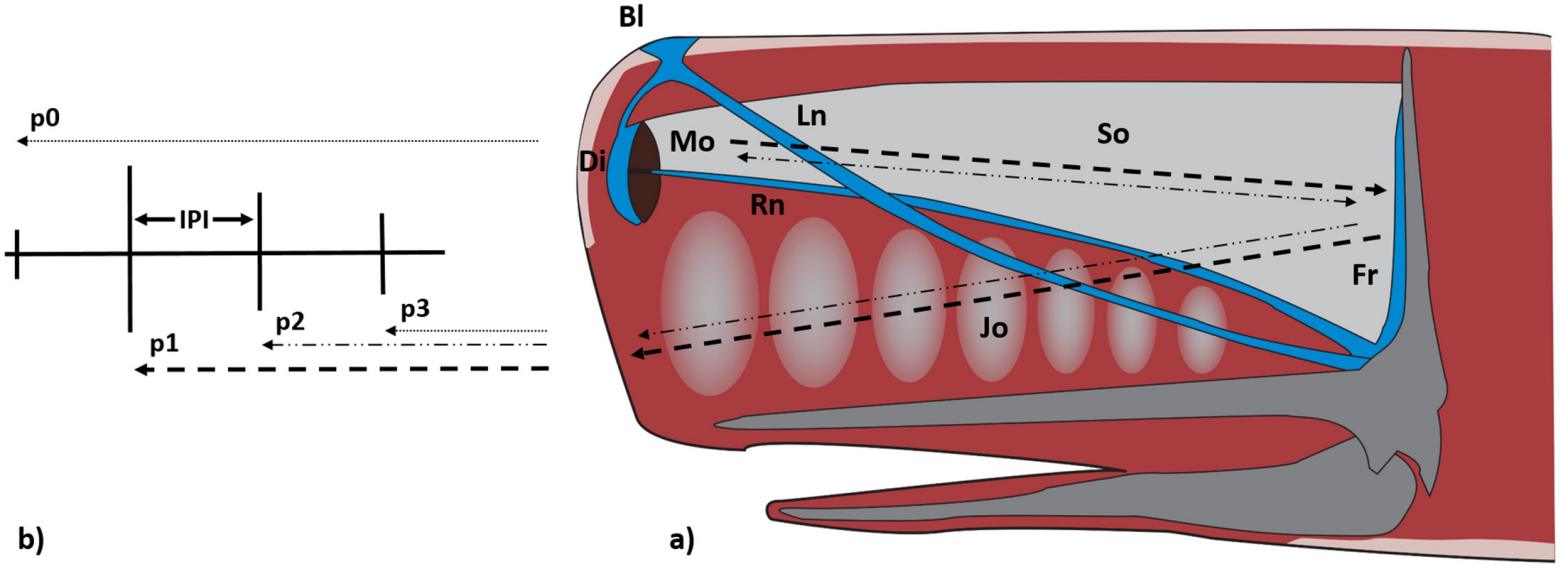
A scheme of the sperm whale’s head and sound production. (a) **Bl**: Blow hole; **Di**: Distal air sac; **Fr**: Frontal air sac; **Jo**: Junk organ; **Ln**: Left naris; **Mo**: Monkey lips; **Rn**: Right naris; **So**: Spermaceti organ. (b) According to the bent horn model, the production of a click generates multiple pulses (p0, p1, p2, p3 etc.).

The sperm whale (*Physeter macrocephalus*, L. 1758) emits sounds underwater to acoustically map the surrounding environment, to search for food and to communicate with conspecifics. The species produces a short multi-pulse signal, named “click”, with time delays of few milliseconds between the pulses [5–7]. The sperm whale’s head houses an enormous skull and a complex system of soft organs (known as ‘spermaceti’ and ‘junk’), air sacs and nasal passages [8, 9]. The nasal passages are asymmetrical, with the right side (Rn) closed and specialized in the production of sound, and the left (Ln) functional for the respiratory system (Fig 1a). The spermaceti organ (So) is a sac filled with a complex mass of oil (spermaceti), the rear of which is in contact with a frontal air sac (Fr). This works as a great ‘sound mirror’ resting on the wide and frontal part of the skull [6, 9]. In the forefront of the skull, the spermaceti organ ends in a pair of black lips of connective tissue (monkey lips, Mo) that produce sounds by way of a pneumatic action [6, 10]. The monkey lips are also connected to the right side of the nasal passage and to the distal air sac (Di), another ‘sound mirror’ at the front end of the head (Fig 1a). The sperm whale produces clicks with duration up to 100 ms, source levels up to 236 dB re 1*μ*Pa (RMS), frequency range to more than 30 kHz with a centroid frequency of 15 kHz [11].

### Stable Inter Pulse Interval and Size Measurement

According to the ‘Bent Horn theory’ [7], after the production by the monkey lips, most of the sound energy reflects on the distal sac and goes backward through the spermaceti organ. During this first reflection, only a weak pulse—named p0—is emitted frontally into the water. It exits from the top of the forehead, containing only 10% of the sound energy generated [12]. The majority of the sound energy travels backward through the spermaceti organ until it reaches the frontal air sac. Here, the powerful pulse is reflected and travels forward through the junk organ to be emitted from its lower part as p1. After the reflection on the frontal sac, a fraction of the pulse energy continues to travel within the spermaceti organ, reflecting back and forth between the two air sacs and exiting from the lower junk (Fig 1a). Therefore, these reflections create repetitive pulses with exponentially decreasing amplitude (p2, p3 etc.) [12] (Fig 1b). Since the p1 and the following pulses are emitted from the same area of the animal’s head (the lower junk) and have the same propagation path, the Inter Pulse Interval between p1–p2, p2–p3 etc. is stable for all clicks emitted by the same sperm whale [13]. The value of this “Stable IPI” varies from about 2 ms to 10 ms, depending on the size of the animal’s head [7], and can thus be used to estimate the total length of the whale [7]. This acoustic size estimation technique has been used to monitor the ecological dynamics of sperm whales around the world [6, 7, 13–24].

## Approach Overview

### Presence of Variable IPIs

The Stable IPI can always be identified within the pulses that compose a click, but its recognition is complicated by the position of the animal and of the receiving sensor [25, 26]. Adler-Fenchel (1980) [14] noted that only a small proportion of recorded clicks was useful for Inter Pulse Interval measurements. In the 1990s, the position of the animal was found to influence the characteristics of the signal received [15, 16]. In off-axis recordings between p0 and p1 there is an intermediate pulse—named p1/2—generated by the reflection of the sound on the frontal sac [25]. Therefore, the time intervals between p0 and p1/2 pulses change in relation to the position of the receiving sensor. The optimal results are obtained recording the animal on-axis, with the receiver located either directly behind or in front of it [25], when the sperm whale starts a deep foraging dive, immediately after the whale flukes up [22]. When the position of the sperm whale is unknown, the Stable IPI can still be estimated by automatically processing a few hundred clicks and averaging the results [13].

### Stable IPI recorded over time

The Stable IPI is considered ‘stable’ for the same sperm whale in dives several months apart, when considering the first minutes of the dive [17, 21]. This value increases over the years, according to the growth rate of the animal [18, 21, 24]. How the IPI may vary with depth is unknown [21]. The sperm whale may dive to more than 2,000 m [27] with descent speeds between 75 and 120 m/min [28]. Small changes in IPI have been hypothesized during the descent phase, due to the effects of the varying environmental conditions (hydrostatic pressure and water temperature) [21, 29]. In 1996, Goold et al. [29] simulated the variations of sound velocity in the spermaceti oil in relation to the depth-dependent environmental conditions, and found a variation of about one tenth (appr. 0.7 ms) for an IPI of 7.2 ms, during a dive to 900 m. In 2002, Madsen et al. [10] observed an almost constant value of the Stable IPI for a dive from the surface to 700 m (variation less than 0.2 ms for a Stable IPI of 3.4 ms).

### The main methods applied

1. The intervals between the pulses of a click can be identified by displaying the waveform of the signal and a high resolution spectrogram. This application requires selection of clicks with a clear multi-pulse structure [14, 15, 20], not contaminated by excessive noise or overlapped by reflections generated by the sea surface. This ‘manual method’ is very accurate but very time consuming, so it is difficult to apply to large datasets.
2. Goold (1996) [16] used cross-correlation and cepstrum analysis of the signal waveform to measure the Stable IPI. The average of results obtained for a great number of clicks was applied to find a reliable value of the Stable IPI [16]. This analysis allows finding the time delay between the consecutive pulses present in a click. The amplitude domain is represented by the independent variable called ‘quefrency’. Data were restricted to clicks recorded within 6 minutes after the sperm whale’s fluke up [17]. Subsequently, the cepstrum analysis was used in several other studies [13, 17, 22].
3. Pavan et al. (1997) [17] developed a software tool that simultaneously shows the real-time spectrogram and the cepstrogram (cepstrum vs time) of the acquired signal. This tool allows real-time visualization of the pulse delays for every well-structured click. This method was useful during surface acoustic surveys, even on clicks with low SNR (Signal to Noise Ratio) [17–19].
4. Teloni et al. (2007) [13] suggested that the Stable IPI can be estimated by processing a few hundred of clicks and averaging their power cepstra. This method can be applied for sperm whales recorded in an unknown orientation.
5. Antunes et al. (2010) [22] compared various Stable IPI estimation methods (manual measurement, waveform averaging, autocorrelation averaging, cepstrum averaging etc.) on a known dataset. Among all the automatic analysis techniques tested, the best performance was obtained by averaging the autocorrelation, but under some conditions, the cepstrum analysis found the Stable IPI where the autocorrelation did not.
6. A tool of PAMGUARD [30], an open source software for passive acoustic monitoring of cetaceans, elaborates the cepstrum analysis of clicks.

### From a Stable IPI to a size measurement

The relationship between the Stable IPI, the size of the sperm whale’s head and the total body length is demonstrated by several works [7, 15, 23, 31]. Gordon (1991) [15] suggested an empirical relationship between the Total Length (TL) of the whale and the Stable IPI, based on estimates of its size through photogrammetry:

$$TL=4.833+1.453\cdot IPI-0.001\cdot IPI^{2}$$ (1)

Gordon tested this formula on recordings of 11 animals that were photographically measured in the Azores and Sri Lanka [15]. Most animals were juveniles; only one was longer than 12 meters. Accordingly, Gordon’s formula is very reliable for measurement of sperm whales with a length ≤ 11 m [10, 23, 32].

In 2011, Growcott et al. studied the relationship between the Stable IPI and photogrammetric measurements of size [23]. A new formula was proposed to estimate the size of sperm whales over 11 m from the Stable IPI value:

TL=1.258·IPI+5.736 (2)

However, new data and techniques are needed to improve the reliability of the acoustic measurement of sperm whale length. Now formulas should also take into account the difference in the head-body allometric relationship between the sexes and changes occurring with age [21].

## Materials and Methods

### Data collection

**Fig 2:**
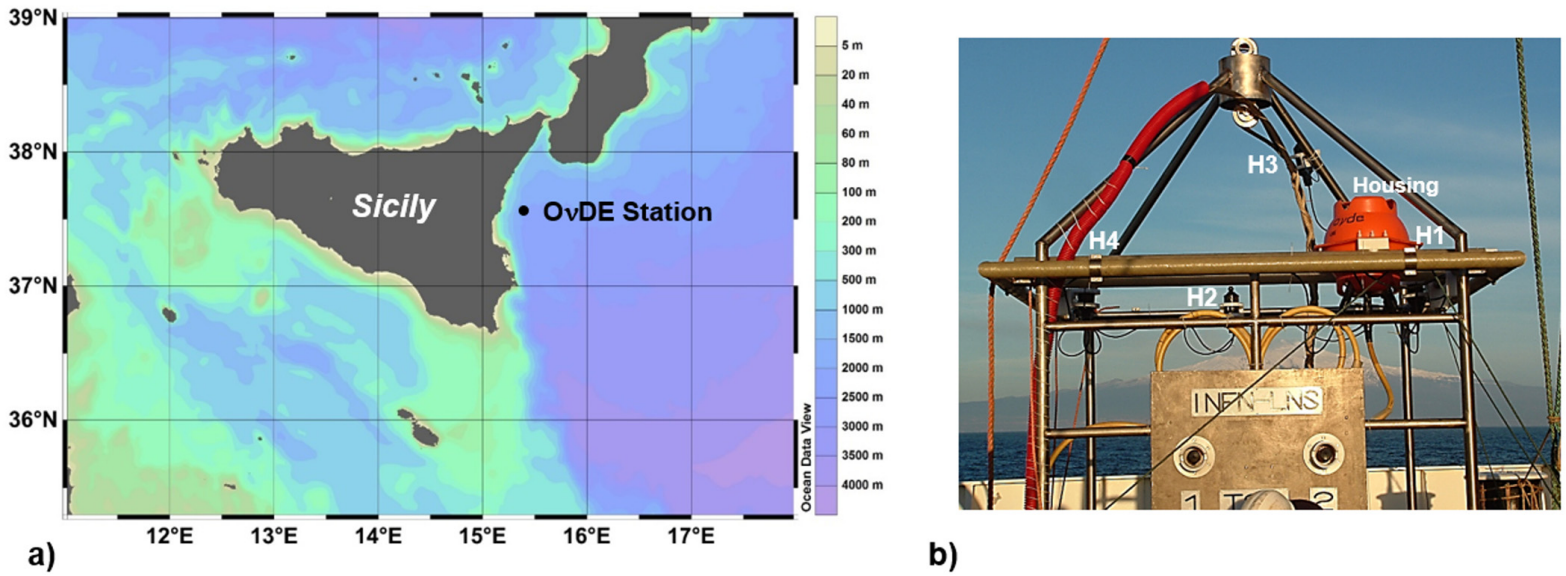
The O*ν*DE cabled seafloor observatory. (a) The site of installation of the O*ν*DE acoustic array (NEMO Test Site—2,100 meters of depth, South-western Ionian Sea). ODV (2012) [45]. (b) Photo of the O*ν*DE frame before the deployment. The hydrophones and electronics housing are labeled (H1, H2, H4, H3, Housing).

The O*ν*DE acoustic antenna, an infrastructure built to test novel technologies for the construction of a deep-sea neutrino telescope [33–35], was installed at the Catania Test site of INFN (Istituto Nazionale di Fisica Nucleare). The O*ν*DE station was in operation in the site from January 2005 to November 2006. The infrastructure consists of a laboratory located in the Port of Catania (Sicily), a 28 km long electro-optical submarine cable laid on the seafloor, and two terminations anchored at a depth of 2,100 m, 25 km from the shore (Gulf of Catania, Ionian Sea). The two terminations are approximately 5 km apart. Both terminations are equipped with electro-optical ROV mateable connectors that allow power feeding from shore to the underwater equipment, and data transmission in real-time over the optical fibers. The North termination is used for geophysics and multidisciplinary experiments of the EMSO (European Multidisciplinary Seafloor Observatory) Research Infrastructure [4, 36, 37]. O*ν*DE was installed on the mechanical frame hosting the connectors of the South termination, located at Latitude 37°32.681’N, Longitude 15°23.773’E (Fig 2a). The O*ν*DE experiment was the first long-term scientific installation for monitoring in real’time the acoustic noise in the deep Mediterranean Sea [38]. O*ν*DE aimed, primarily, at studying acoustic noise for future experiments on neutrino detection [39], but it had significant follow-ups in bioacoustics [40]. The O*ν*DE acoustic antenna is made of four piezoelectric omnidirectional hydrophones (RESON TC4042-C) displaced in a tetrahedral shape. One hydrophone (H3) is installed on the top of the frame, placed approximately at about 3.5 metres above the seafloor. The other three sensors (H1,H2,H4) are installed at about 2.5 metres from the seafloor (Fig 2b). The hydrophones were certified by RESON [41] to operate at 2,500 m depth with a sensitivity of about -193 dB re V/*μ*Pa, in the frequency range from 100 Hz to 40 kHz (coupled to a 20dB preamplifier made by RESON). Hydrophone data were sampled underwater using a couple of stereo ADC converters CS5396 from Cirrus Logic [42]. Sampling frequency of 96 kHz with 24 bit quantization were used; the two stereo streams were kept synchronized by a common clock. Two electro-optical modems (data transmitter—ECT-100E-VT-M1 model) [43] provided the transmission of the digital audio streams through the submarine electro-optical cable. On shore, two soundboards RME DIGI96/8-PAD were used to acquire in real-time the data streams 24/24h. The software WaveinRecorder, developed by CIBRA [44], was interfaced to the hardware devices for the data acquisition. The software kept synchronized and joined the two data streams to provide data storage and real-time display. Due to storage size limitations, twenty-four files per day (each containing 5 min of data, taken every hour) were stored. Each file contains the four audio channels (32 bit format, size of 450 MB per file). A total of about 5,000 recordings (5min/hour) were acquired during the year 2005. The dataset analysed in this work consists of 2,128 recordings, selected among 93 days of acquisition in which the presence of sperm whale clicks was documented for, at least, 1 file per day through the pre-survey of the data (spectrogram analysis and listening). To verify the accuracy of the automatic method we applied it to a small dataset from 2006 (15 recordings) that has been accurately analysed manually. The data in this paper were taken from the hydrophone H3, installed on the top of the frame.

### The algorithm to identify Stable IPIs in the O*ν*DE dataset

**Fig 3:**
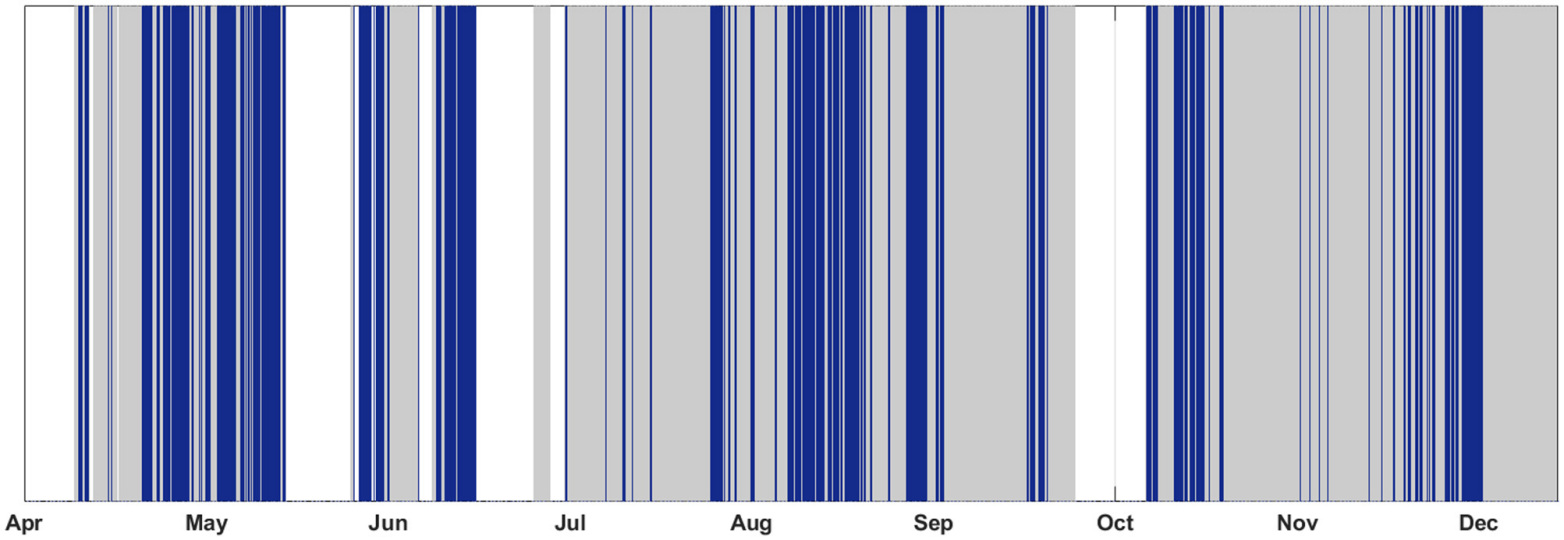
Acoustic Presence of Sperm Whales from April 1^st^ to December 31^st^ 2005. The days of data acquisition in 2005 are shown in gray (from April 1^st^ to December 31^st^). The days with acoustic presence of sperm whales recorded in at least 1 file are shown in blue.

Initially, in order to reveal the presence of cetacean sounds, the acquired data were analysed using the software SeaPro [44], which allows the visual investigation of the spectrograms and, when required, the simultaneous listening of the recordings. The acoustic monitoring of sperm whales provided the first results on the seasonal presence of the species in the area from April 1^st^ to December 31^st^ 2005 (Fig 3). The results highlighted the presence of sperm whales in about 50% of the days in which recordings were acquired [46]. Then the whole dataset was investigated through spectrogram analysis and listening. The present study required the development of an automatic detection method to extract the clicks to be analysed for the Stable IPI measurement.

#### Stable IPI measurement

**Fig 4:**
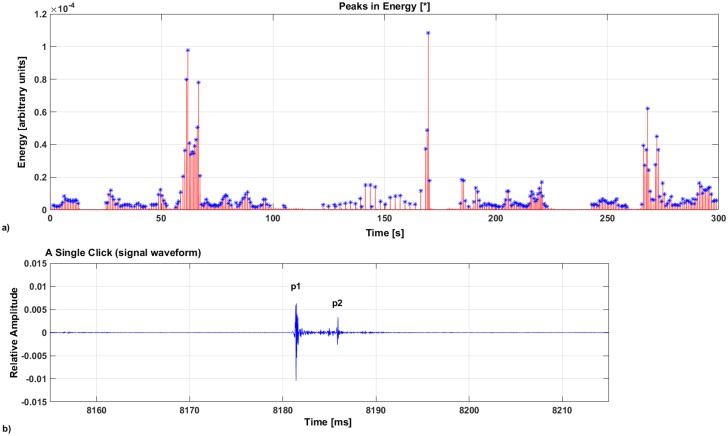
The detection of clicks emitted by sperm whales. (a) The detection of short pulses in the energy of the signal (Butterworth filter, bandpass 3–16 kHz). (b) A single click extracted; the pulses p1 and p2 are shown in the signal waveform. p0, p1/2 and p3 are not visible in the figure.

A new algorithm was developed in MATLAB environment to automatically select the recordings with a clear structure of the sperm whales’ clicks, in order to estimate the Stable IPI values. The algorithm identifies and selects the Stable IPI (p1–p2, p2–p3, etc.) from the Variable IPIs (p0–p1, p0–p1/2). The detection of the clicks occurs applying a band-pass filter from 3 to 16 kHz (Butterworth filter). An adaptive threshold, calculated on the median value of the total sound energy in the 5-min recording, is then applied. The threshold (T) allows the selection of short acoustic events in the frequency band 3–16 kHz (Fig 4a).

$$T=500\cdot (median\left(x^{2}\right))$$ (3)

where *x* is the amplitude of the acoustic signal acquired in 5-min recording.

The algorithm selects the amplitude peaks and, for each detection, a time frame of 60 ms is extracted (Fig 4b). This time interval was chosen in order to extract and analyse the whole series of multiple reflections of the click, generated inside the sperm whale’s head. The cepstrum analysis is then performed to find the Stable IPI value within the signals extracted [13], both in amplitude and in energy (squared amplitude).

$$C=|FFT^{-1}(log|FFT\left(\left|x^{2}\right|\right)\left|\right)|$$ (4)

where *x* are the samples extracted.

The results obtained in a 5-min recording for all computed cepstra were averaged. Following Teloni et al. (2007) [13] and Antunes et al. (2010) [22], a reliable measure of the Stable IPI was considered by averaging the results for, at least, 50 clicks within the same 5-min recording.

M=mean(C) (5)

where *M* is the average of the cepstra (*C*) applied to the signals extracted.

**Fig 5:**
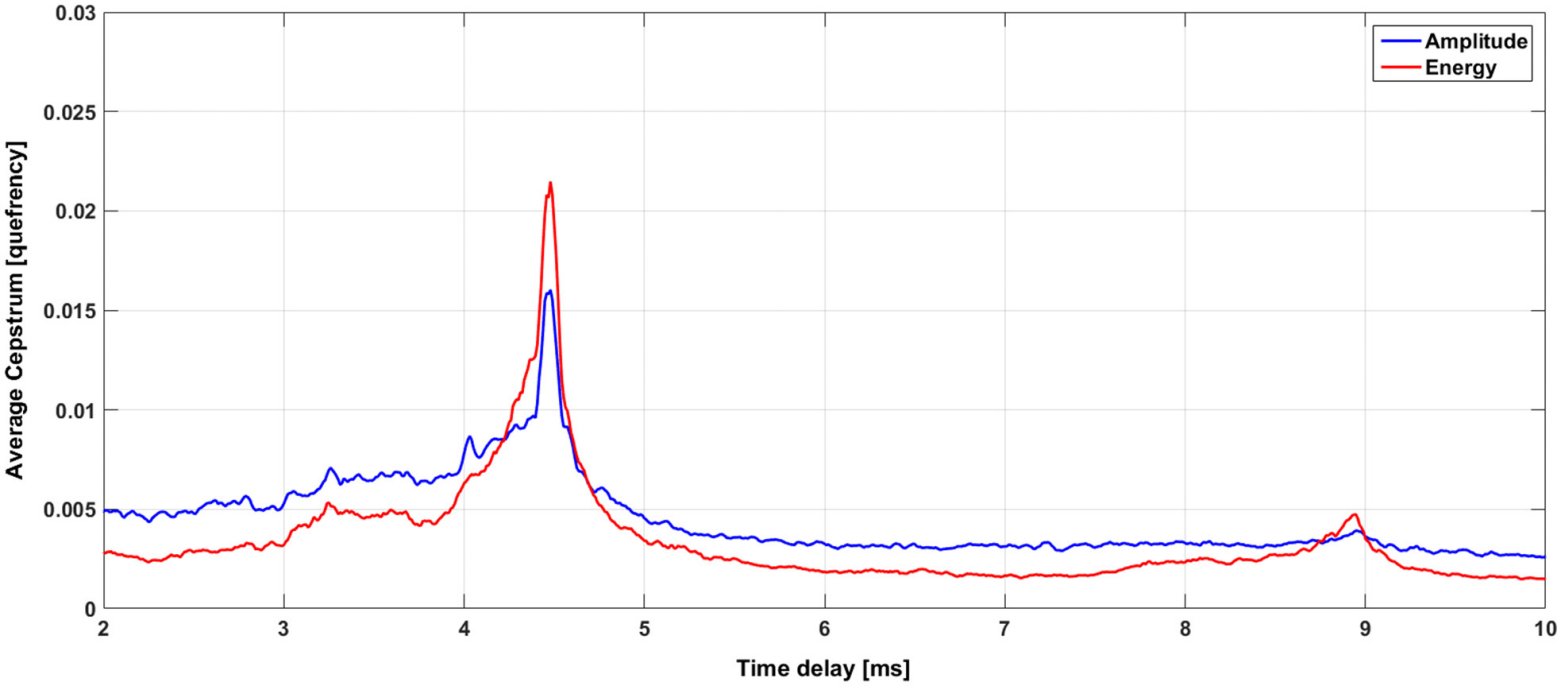
The average of the cepstrum analysis for the clicks selected in a 5-min recording. A Stable IPI of about 4.5 ms is shown as a time delay between the pulses. The cepstrum analysis is applied to the clicks identified (Fig 4), using both the amplitude (blue line) and the energy (red line) of the signal.

**Fig 6:**
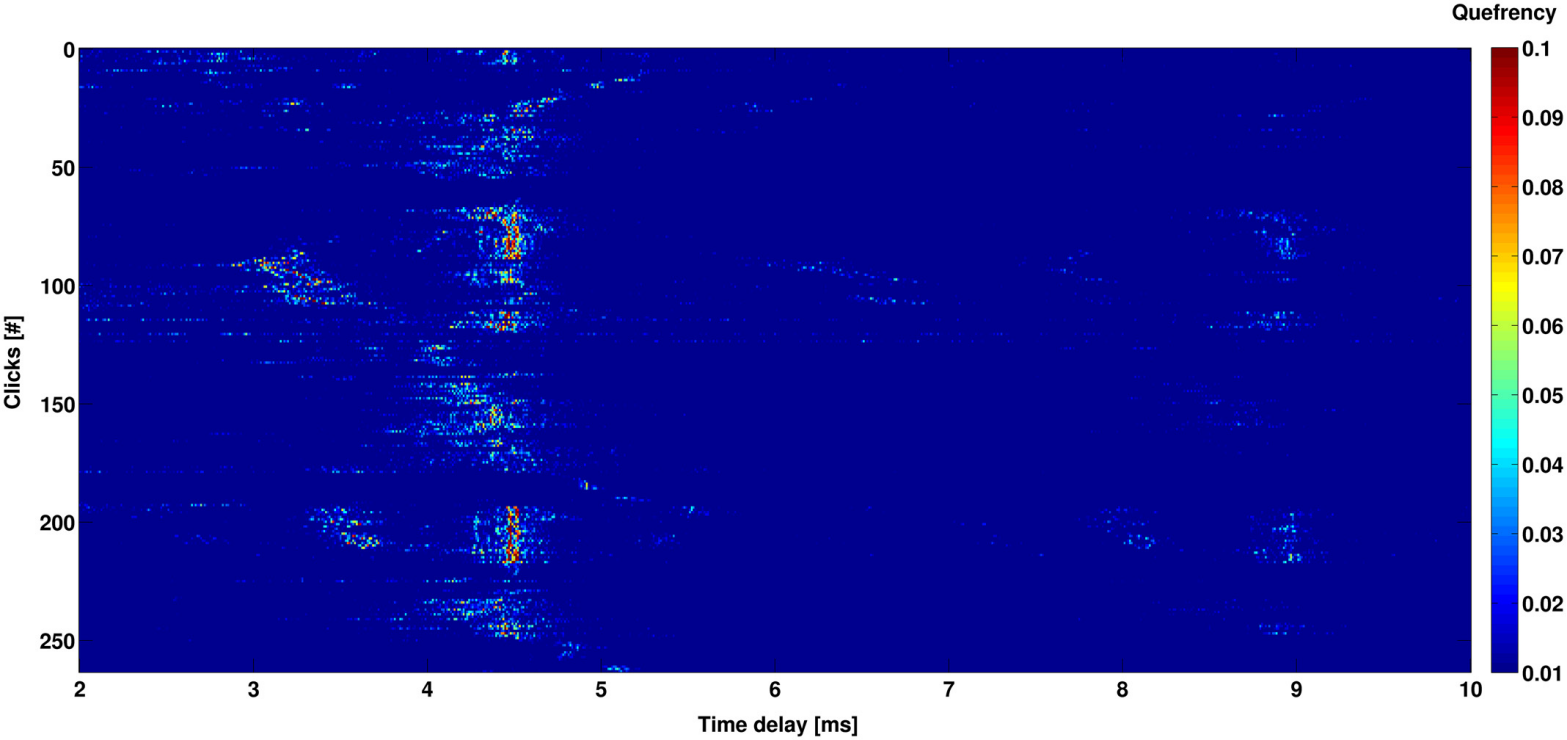
The cepstral peaks identified in a 5-min recording. The figure shows the cepstra values found for all the clicks detected (Figs 4–5). The value for each click varies as a function of the Inter Pulse Interval found, applying the cepstrum analysis to the energy of the signal extracted. A Stable IPI of about 4.5 ms is present and for some clicks the time interval p1–p3 (about 9 ms) is also visible.

The search for the cepstral peaks (that reveal equally spaced pulses) was conducted in the delays from 2 to 10 ms (Fig 5). In the research of the Stable IPIs, a small variation of the cepstral peak during the five minute recordings was considered, following the results obtained by Madsen et al. (2002) [10]. The algorithm applies a threshold to the median of the cepstra average and the Stable IPI values were selected within time intervals of 0.3 ms. The error on the single measurement of a Stable IPI has been calculated ± 0.05 ms. This value has been computed as the square root of the sum in quadrature between the sampling time accuracy and the 3 *σ* of the cepstrum peak distribution, used to evaluate the stable IPI. Cepstral peaks of consecutive clicks are shown in Fig 6. In order to verify the reliability of the results obtained, a pre-analysed subset of O*ν*DE (October 2006) has been also processed. For 15 recordings with sperm whale’s clicks, a comparison with the results obtained through the manual measurement of the Stable IPIs was developed. The Stable IPIs were manually measured by using the signal waveform and averaging the results for at least 50 clicks per recording.

#### Size estimation

The equations of Gordon (1991) [15] and of Growcott et al. (2011) [23] were used to transform the Stable IPI value into sperm whale size. Rice’s curves of growth were used to attribute the sex of the sperm whale recorded [47] and three size classes and the relative sexual maturity were considered [48]:

- Immature Male or Female: *TL* < 9 m;
- Adult female or Juvenile Male: 9 m < *TL* < 12 m;
- Adult Male: *TL* > 12 m

The availability of 5-min/hour recordings did not allow us to firmly confirm the detection of the same animal in consecutive files. For each recording, the presence of more than one animal clicking together and similar in size (Stable IPI different for less than 0.3 ms) was not distinct.

### Ethics Statement

The O*ν*DE deep-sea cabled infrastructure was deployed off the East Sicilian coasts with all regular authorisation issued from civil and military authorities, involved at National and Regional scale: Marina Militare Italiana, Autorità Portuale di Catania, Ministero dell’Ambiente and Direzione Marittima di Catania. The sperm whale (*Physeter macrocephalus*—Mediterranean subpopulation) is included in the IUCN (International Union for the Conservation of Nature and Natural Resources) Red List of Threatened Species and listed as “endangered” [49]. The Passive Acoustic Monitoring (PAM) of sperm whales using cabled deep-sea observatory does not require approvals and the techniques applied do not imply any interference or contact with the studied animals.

## Results

### The Stable IPIs identified in the O*ν*DE Dataset

**Fig 7:**
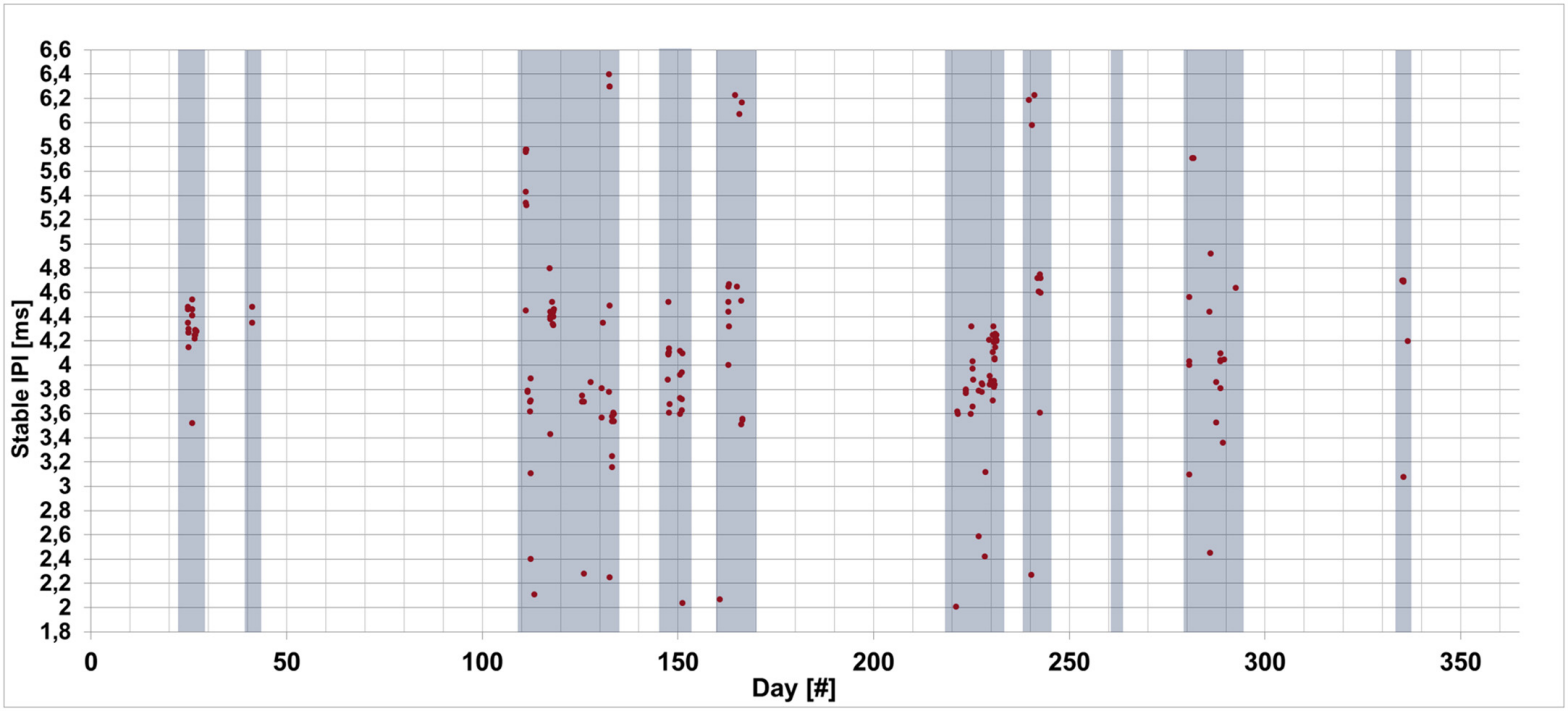
The Stable IPIs identified within the O*ν*DE dataset from 24^th^ January to December 2^nd^ 2005. The Stable IPI identified are shown by red dots. Highlighted in blue, the 93 days analysed with the automatic algorithm. The acoustic presence of sperm whales was documented by spectrogram analysis and listening for all the recordings where Stable IPIs were measured. The error on the single measurement is ± 0.05 ms, calculated as the square root of the sum in quadrature between the sampling time accuracy and the 3 *σ* of the cepstrum peak distribution, used to evaluate the Stable IPI.

Over 5,000 audio files (each lasting five-minutes) were recorded by the O*ν*DE station in 2005, the dataset analysed in this work consists of 2,128 recordings. In 1,357 of these (63.8%), the algorithm identified short acoustic events in the frequency band from 3 to 16 kHz. Within this batch of recordings, the cepstrum analysis applied to the energy of the signal resulted in the measurement of 183 Stable IPIs in 156 files. The range of the Stable IPIs identified is from 2.1 to 6.4 ms (Fig 7). In 26 recordings, two different Stable IPIs were found and only in a single file three values were selected (resolution of 0.3 ms). The presence of more than one animal clicking together and similar in size was not distinct. The new application of the cepstrum to the energy of the signal performed better than the same analysis applied to the waveform amplitude, that produced results in only 16 files. The accuracy of the automatic method was verified on an independent 15-file subset, recorded in 2006, by manual measure of the IPIs (Table 1). The software SeaPro, developed by CIBRA [44], was used in order to reveal the presence of sperm whale sounds through spectrogram visual analysis and listening [46].

**Table 1:** A comparison between the Stable IPI obtained applying the manual and the automatic methods.

**Sperm Whale**	**Manual [ms]**	**Automatic [ms]**
SW1	4.47	4.48
SW2	5.08	5.05
SW3	5.13	5.05
SW4	5.13	5.07
SW5	5.12	5.11
SW6	5.77	5.75
SW7	5.75	5.73
SW8	5.77	5.72
SW9	5.75	5.73
SW10	5.13	5.11
SW11	5.17	5.08
SW12	5.03	5.02
SW13	5.19	5.18
SW14	5.00	5.02
SW15	4.91	4.92

Mean values of Stable IPI measured in 15 files of a known sub-set (O*ν*DE station, 15 recordings, October 2006). The error associated to the manual technique is ± 0.03 ms, being measured on selected louder clicks. For the automatic method the error on the single measurement is ± 0.05 ms.

### Size measurement of the detected sperm whales

**Fig 8:**
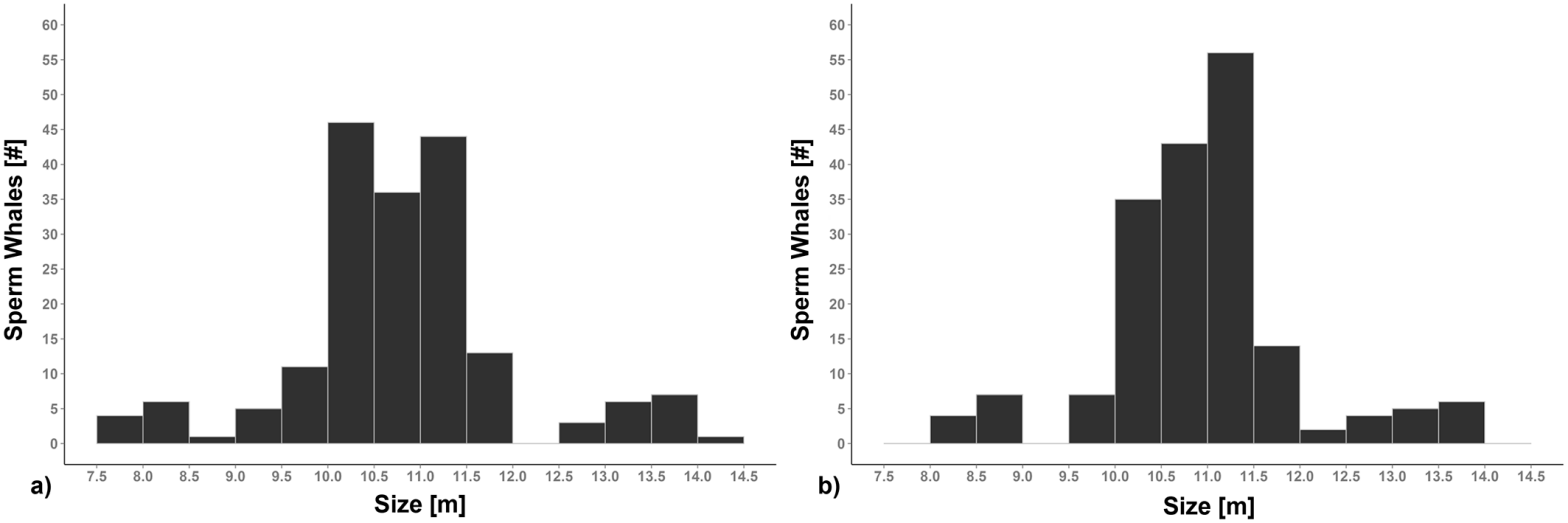
The size of the sperm whales grouped in classes of 50 cm. (a) The results obtained using Gordon’s formula. (b) The results obtained using Growcott’s formula.

From the Stable IPI values selected applying the described technique, the sperm whales’ size was derived using Gordon’s formula [15] and Growcott’s formula [23]. The results obtained using the two equations showed statistical difference (Sign Test: Z = 10.45, P<0.001; Wilcoxon test: Z = 8.45, P<0.001) (Fig 8).

**Fig 9:**
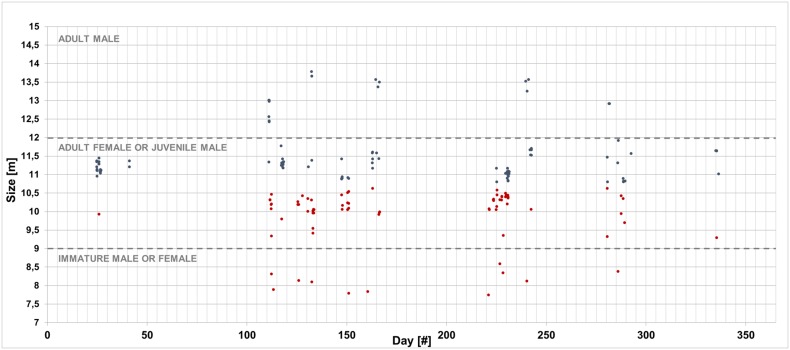
The size of the sperm whales detected from January 24^th^ to December 2^nd^ 2005. The size of the sperm whales with a Stable IPI ≤ 4 ms are represented using the equation of Gordon (red dots) [15]; the equation of Growcott et al. (2011) [23] is instead used only for the animals with a Stable IPI > 4 ms (about 11 m in length) (blue dots). Knowing the size of the animals recorded, the sex and sexual maturity are hypothesized.

For a greater reliability of the results, the equation of Gordon (1991) [15] was used for the measurement of the size of sperm whales with a Stable IPI ≤ 4 ms and the equation of Growcott et al. (2011) [23] for the animals with a Stable IPI > 4 ms (about 11 m in length). Several sperm whales, ranging from about 7.5 m to 14 m, were measured and the sex/sexual maturity of the animals was also hypothesized (Fig 9).

## Discussion

The Mediterranean sperm whale is a sub-population genetically distinct from the Atlantic conspecific [50]. The Mediterranean population is considered to be composed of less than 2,500 mature individuals and it is listed as endangered in the Red List of Threatened Species of IUCN [49]. Information about a clear population structure is not available for the Mediterranean Sea [51]. In the last decades, several factors influenced the high level of mortality of this species [52] and such a decline will continue without effective conservation and management actions [53]. A regular monitoring of sperm whale abundance, distribution and habitat throughout the different regions of the Mediterranean area should provide requirements to protect the population in the entire maritime basin [53]. Recently, a comparison between the photo-ID of sperm whales in the Western and Eastern districts of the Mediterranean Sea confirmed the evidence of inter-basin movements of the species [54]. Therefore, in this scenario, the Ionian Sea could be a preferential hub for the migration of the species within the Mediterranean basin [46, 54, 55]. The ecological dynamics of the sperm whales occurring in the area continue to be little known and only fragmented information is available. In the 1940s, groups of sperm whales moving across the Strait of Messina were documented several times [56]. In 2003, the results of a boat based survey financed by IFAW (International Fund for Animal Welfare) showed the presence of sperm whales in the Ionian Sea decreased to a few animals [57]. In 2005, the O*ν*DE experiment recorded sperm whales in about the 50% of the acquisition days [46]. From 1991 to 2002, the size of several sperm whales recorded during the summer months in different areas of the Mediterranean Sea was acoustically measured [18–20]. The presence of the species was reported in the whole basin and a range of variability in terms of size was reported (from about 8.5 to 14 metres) [17–20]. The animals recorded were considered both young and adult specimens. In detail, in the Ionian Sea the species seemed to be present in different size categories [58, 59]. In this work, an assessment of the size distribution—based on acoustic measurements—of several sperm whales recorded during a long deep-sea monitoring in the Ionian Sea is given. A dataset of about 2,100 recordings (5 min/h each) was automatically analyzed. The acoustic presence of the species was confirmed with spectrogram analysis and listening of the recorded data. A number of sperm whales, recorded in 2005, was characterized in size (from 7.5 m to 14 m). The size category most represented was from 9 m to 12 m (adult females or juvenile males) and specimens longer than 14 m (old males) seemed to be absent in the area. As to the measurement of the Stable IPI, a comparison with the manual method of analysis did not show a significant difference. A better performance in the measurement of the Stable IPI was possible analysing the energy of the clicks rather than just the waveform amplitude. The new implementation of the cepstrum analysis resulted more efficient than traditional methods and allows the unsupervised processing of huge amount of acoustic data provided by long-term acoustic monitoring platforms.

## Conclusions

In the Mediterranean Sea, the sperm whale is subject to multiple anthropogenic pressures and the effects on the entire population are still unknown. Crucial data could come with the spreading Passive Acoustic Monitoring (PAM) activities, but also from off-shore visual assessments (photo-ID) and other technologies such as DTAG [10], radio-tracking and Gliders [60]. A correlation between the photo-ID and the acoustics could allow to study the population structure and the growth rate of the sperm whales through a non-invasive method, considering that the Stable IPI increases over the years in relation to the growth of an individual [18, 21, 24]. The huge amount of data acquired by the deep-sea cabled O*ν*DE observatory allowed to increase our knowledge on the ecological dynamics of the sperm whale in the Central Mediterranean Sea. The capability to acquire data for a long time and from seafloor multidisciplinary station provides advantages and new opportunities to study this deep-diver cetacean species.
